# Low-cost materials for swine wastewater treatment using adsorption and Fenton’s process

**DOI:** 10.1007/s11356-023-29677-1

**Published:** 2023-09-18

**Authors:** Eva Domingues, João Lincho, Maria J. Fernandes, João Gomes, Rui C. Martins

**Affiliations:** CIEPQPF—Chemical Engineering Processes and Forest Products Research Center, Department of Chemical Engineering, University of Coimbra, Rua Sílvio Lima, Polo II, 3030-790 Coimbra, Portugal

**Keywords:** Swine wastewater, Natural adsorbents, Fenton, Low-cost catalysts, Wastewater treatment

## Abstract

Untreated swine wastewater (SW) discharge leads to serious consequences such as water quality decreasing related to eutrophication and proliferation of harmful algae containing cyanotoxins, which can cause acute intoxication in humans. The use of untreated pig farming effluent as fertilizer can lead to the accumulation of polluting compounds. Biological treatments can degrade organic matter but have the disadvantage of requiring large areas and high retention times and demonstrating low efficiencies in the degradation of refractory compounds such as pharmaceutical compounds. In this ambit, the performance of four low-cost materials was evaluated for treatment of a swine wastewater using physical–chemical processes such as adsorption and Fenton’s process. The tested materials are two natural resources, red volcanic rock from Canary (RVR) Islands and black volcanic rock (BVR) from Azores, and two industry residues, red mud (RM) and iron filings (IF). Among the tested materials, only IFs are catalytically active for Fenton’s peroxidation. Still, RVR, BVR, and RM were efficient adsorbents removing up to 67% of COD. The combination between adsorption followed by Fenton’s process using IF as catalyst showed interesting results. When RM is applied as adsorbent in the diluted effluent, it was able to remove 67% and 90% of COD for adsorption and adsorption followed by IF Fenton, respectively. At those conditions, the resultant treated effluent accomplishes the requirements for direct discharge in the natural water courses as well as the parameters for water reusing.

## Introduction

The consumption of swine origin products is increasing over the years (Oliveira et al. 2020; Fao 2020), in which the pork is the second most consumed meat (Nagarajan et al. 2019) worldwide. To ensure the food market needs, the swine industry is expanding rapidly (Garcia et al. 2020) raising about one billion pigs every year (Cheng et al. 2020) corresponding to about 102 million tons of pork meat (López-Pacheco et al. 2021). This industry produces large wastewater residues with high content of organic pollutants (Chen et al. 2021), and it is responsible for gas emissions estimated of over 7 billion kg of nitrogen per year, between 2010 and 2017 (Cheng et al. 2020). The wastewater resulting from swine industry is complex and may lead to environment concerns if released in nature without the correct treatment (Nagarajan et al. 2019; Folino et al. 2020).

The swine wastewater is mainly composed by feces, urine, undigested food, water spillage, hormones, antibiotics, and pathogenic organisms (Folino et al. 2020; Zhang et al. 2019), which means that it is expected to present several contaminants in their constitution, namely, fatty acids, nitrogen, phosphorous, heavy metals, antibiotics, suspended solids, and fecal coliforms (Cheng et al. 2020; Emerick et al. 2020; Garcia et al. 2020; Zhang et al. 2016).

Swine wastewater presents high levels of ammonium, estimating a total production of 16 kg N/animal/year (Nagarajan et al. 2019). The nitrate is considered toxic to humans and to the environment and can lead to health problems when ingested, namely, the methemoglobinemia, also known as “blue baby syndrome” (Prokhorova et al. 2021). The antibiotics usually consumed by the pigs are excreted by the animals and are difficult to remove from wastewater (Chan et al. 2022). Moreover, the presence of virus and protozoan agents in swine wastewater can be a source of diseases for animals and even humans (Garcia et al. 2020). Therefore, it is important to ensure the correct treatment and decontamination of this type of wastewater to reduce the water contamination and to prevent human health problems.

In general, this type of effluent is treated with the conventional biological methods. However, these are not totally efficient to reach the legal limits for discharge (Emerick et al. 2020; Gomes et al. 2021). Fenton process and adsorption show potential on the abatement of the organic content and bacteria when applied to the treatment of swine wastewater (Qian et al. 2020; Domingues et al. 2021). Domingues et al. (2021) applied the homogeneous Fenton process integrated with coagulation proving great impact over the abatement of organic content and toxicity reduction. However, an additional step of coagulation and adsorption was needed to remove the dissolved iron and iron sludge. Regarding adsorption, Kannan and Parameswaran (2021) studied the ammonia adsorption and recovery from swine wastewater permeate using naturally occurring clinoptilolite. The authors managed simultaneous removal of P from real and synthetic swine wastewaters. The removal of ammonia–nitrogen and phosphate from simulated swine effluents using modified zeolite was also studied by Huang et al. (2014). The authors used as adsorbent modified natural zeolite by magnesium salts; the Mg^2+^ released from the adsorption process served as a source of magnesium in struvite crystallization.

The advanced oxidation processes can be suitable solutions regarding the swine wastewater treatment, since the degradation of pollutants is promoted by hydroxyl radicals that could lead to mineralization (Oller et al. 2011; Gomes et al. 2017); among them, the Fenton’s process can be an interesting option (Bautista et al. 2008; Ochando-Pulido et al. 2017). This technology generates hydroxyl radicals by the interaction between iron and hydrogen peroxide, usually at acidic pH (Duesterberg et al. 2008). Moreover, other metals such as Cu, Ce, Mn, or Ag can replace iron (Hussain et al. 2021).

This study aims to evaluate the performance of four low-cost materials as adsorbents and heterogeneous catalysts in heterogeneous Fenton process, for the treatment of a swine wastewater. The tested materials are the red volcanic rock from Canary (RVR) Islands, black volcanic rock (BVR) from Azores, red mud (RM), and iron filings (IF). The volcanic rocks are abundant and available in nature, while the red mud and the iron filings are industry residues. The use of these materials improves the circular economy while at the same time motivates the use of natural materials for environmental applications.

Red mud is a residue rich in iron and is formed during the aluminum oxide production by a process known as Bayer process. Every year, about 66 million tons of red mud waste is generated (Domingues et al. 2019). IFs are residues from metal-processing industry being composed by zero valent iron and iron oxides among other metals. Besides, the red and the black volcanic rock also present iron in their constitution (Martins et al. 2013; Gomes et al. 2018), what makes them an expectable good performance catalyst for Fenton’s oxidation process. These materials were never applied as adsorbents for the wastewater treatment. RM and the volcanic rocks have a high metallic content as well as high specific surface area that may enhance contaminant coagulation and adsorption (Domingues et al. 2019; Martins et al. 2013; Gomes et al. 2018).

To the best of our knowledge, this is the first time that these materials are used for the treatment of real swine wastewater.

## Material and methods

### Wastewater preparation and characterization

The raw swine wastewater was collected from a pig farm located in the center region of Portugal. The swine wastewater used in the experiments was obtained by diluting the raw wastewater with distilled water in a ratio of 1:12.5 (8% vol.). This dilution was made to simulate the washing step in pig farming, which did not happen at the effluent collection time (Domingues et al. 2021, Gomes et al. 2021).

The swine wastewater was coagulated using 0.1% of polydiallyldimethylammonium chloride (PolyDADMAC) as described in the previous study of Domingues et al. (2021), also considering the same sedimentation period (1 h). The diluted and coagulated effluents were characterized considering the chemical oxygen demand (COD), biochemical oxygen demand at day 5 (BOD_5_), total solids (TS), total Kjeldahl nitrogen (TKN), total phosphorous, and pH (Table 1).

**Table 1:** Characterization of diluted and coagulated swine wastewater effluent

Parameter	Diluted effluent	Coagulated effluent
COD/[mgO_2_/L]	2980–3150	821–1050
BOD_5_/[mgO_2_/L]	288–527	40–75
TS/[mg/L]	2598–4340	838–1060
TKN/[mg/L]	245–273	112–140
Total phosphorous/[mg/L]	72–80	56–67
pH	7.4–7.6	7.4–7.6

### Reagents and catalysts

Four different low-cost materials (RM, RVR, BVR, and IF) were tested for the swine wastewater treatment. The red mud was supplied by a Greek aluminum industry, while the red and black volcanic rocks were collected in the Canaries and Azores islands, respectively. The iron fillings were acquired by unmaking iron wastes. All the materials, except the iron fillings, were sieved using a 0.105-mm sieve.

RM and the volcanic rocks’ elemental characterization was carried by flame atomic absorption spectrophotometry (Perkin Elmer 3300) after acid digestion. The black and the red volcanic rock characterization was previously carried by Gomes et al. (2018) and Martins et al. (2013), respectively. Moreover, the red mud was also characterized in the work of Domingues et al. (2019). In these works, different properties such as metal content, SEM images, EDS spectra, XRD, BET analysis, and pore dimensions were analyzed. The catalyst’s pH zero point of charge (pH_zpc_) was determined considering the method described in Rivera-Utrilla et al. (2001)’s work, for both volcanic rocks and for the red mud. The effluent pH was adjusted using H_2_SO_4_ 6 M solution and NaOH 10 M solution, acquired from Panreac. The Fenton reaction used hydrogen peroxide (H_2_O_2_, 33% w/V) acquired from Panreac.

### Analytical methods

The chemical oxygen demand (COD) was carried considering the standard method 5220D (Greenberg et al. 1985). The COD calibration curve was prepared using potassium hydrogen phthalate (KHP) (Panreac). The digestion occurred at 150 ºC in the thermo-reactor (ECO25, Velp Scientifica) during 2 h, and after it, the samples were left to cool in the dark at room temperature during 1 h. The absorbance values were measured using a photometer (HI83399 COD photometer, Hanna instruments).

The biochemical oxygen demand (BOD_5_) was evaluated using a respirometry measurement by monitoring the pressure of a closed vessel at constant temperature of 20 ºC, after 5 days. This procedure was monitored by a controller (OxiTop® OC100, WTW) that measures, registers, and processes the pressure that is obtained by a measuring head (OxiTop®-C, WTW) that is equipped in each vessel.

The zeta potential analysis was carried by electrophoretic light scattering in a Malvern Zetasizer Nano ZS (ZEN3600, Malvern Instruments Ltd, United Kingdom).

The catalyst leaching effect was evaluated by flame atomic absorption spectroscopy (Analytic Jena-ContrAA300), in which the experiment final samples were analyzed to determine dissolved iron.

The total solids were determined by drying the effluent samples in an oven at 105 ºC until a constant weight is reached. The samples and effluent pH were measured using a pH meter (HANNA instruments).

Nitrogen was quantified the Kjeldahl method (DIN EN 13342, 2001) using the DKL and UDK units from VELP Scientifica. Total phosphorus was determined using UV–Vis spectrophotometry (PG Instruments T6) at 650 nm by EPA method 365.3.

### Experimental procedure

#### Adsorption experiments

The adsorption experiments were carried in small plastic cups of 100 mL, which were filled with 40 mL of effluent and with the amount of material under test (0, 2.5, 5, 7, and 10 g/L). The cups were placed in a rotatory shaker at 13 rpm and at room temperature (20 ºC).

After different times of agitation (5, 15, 30, and 60 min), the cups were removed from the agitator and left to settle during 60 min. After this sedimentation time, the supernatant was separated from the sedimented solids. These adsorption experiments were carried out at pH = 3 ± 0.2 and at the effluent’s natural pH (7.4 ± 0.2).

#### Fenton’s reaction

After adsorption, it was introduced 80 mL of the (adsorbed) wastewater supernatant into the cups with the iron filings, the pH was confirmed and adjusted if needed, and the right amount of H_2_O_2_ was added to start the reaction. The Fenton reaction was stopped after the desired time by adjusting the solution pH to 11 with the addition of NaOH. The Fenton experiments were carried in a rotatory shaker at 13 rpm in 100-mL plastic reactors.

## Results and discussion

The Fenton’s process has been widely studied in the treatment of various industrial effluents. Swine wastewater is considered a biodegradable effluent, and therefore, the use of advanced oxidation processes has not played a major role for this wastewater. However, when considering the need for a large area for biological treatments and the fact that this effluent may contain compounds that are not biodegradable, such as antibiotics, these processes gain importance.

The great disadvantage of the Fenton process is the formation of iron sludge; therefore, in this study, it was decided to evaluate heterogeneous catalysts, residues, or natural materials present in the environment for the treatment of the real effluent. The waste catalysts studied were IF and RM, while the natural materials were BVR and RVR. According to what has already been done in the study by Domingues et al. (2021) with homogeneous Fenton process, the real effluent first undergoes a coagulation process with polyDADMAC and only after, the heterogeneous Fenton process will be applied.

In an initial stage, some preliminary experiments indicated that RM and the volcanic rocks could lead to higher removal rates by adsorption than the values obtained in the Fenton experiments. Therefore, adsorption experiments were carried out to understand these materials’ adsorption capacity over the organic matter removal, and only the iron fillings were further evaluated for the Fenton’s oxidation process.

In this work, firstly, the materials’ characterization is presented and discussed, followed by the results related to the adsorption results of RM, RVR, and BVR and the Fenton results with the IF. Also, different experiments involving other conditions are presented.

### Material’s characterization

The zeta potential is an important parameter to evaluate the stability and tendency to floc formation, that is, a good indicator of interactions between particles and, therefore, of the stability of colloids and particles in solution. This indicates the potential required to break the protective film of ions that surround the particle in the solution. The addition of the coagulating agent acts on destabilization, which is the reduction of the zeta potential, as the electrolyte reduces the repulsive forces allowing the action of the attractive Van der Waals forces to promote aggregation and consequently the formation of flocs. That is, the addition of the coagulant will bring the zeta potential closer to zero and decrease the repulsive forces. The zeta potential is undoubtedly an explanatory variable of coagulation mechanisms and can also be used to determine the optimal dose of coagulant. The analysis of these two variables, the catalyst’s pH zero point of charge (pH_zpc_), and the effluent zeta potential (Ψ) were carried to understand the catalyst’s adsorption capacity.

Regarding the effluent charges, the zeta potential was also determined for the diluted and coagulated effluent. The catalyst’s pH_zpc_ and the wastewater zeta potential results are present in Tables 2 and 3, respectively.

**Table 2:** Catalyst’s pH_zpc_

Catalyst	pH_zpc_
Red volcanic rock	7.8
Black volcanic rock	5.7^a^–6.8
Red Mud	13^b^

^a^Determined in Gomes et al. (2018)

^b^Determined in Domingues et al. (2019)

**Table 3:** Effluent’s zeta potential

Wastewater	pH	Zeta Potential (Ψ)/[mV]
Diluted swine wastewater	3	− 10.2
	5	− 17.3
	7	− 19.0
	10	− 25.0
Coagulated swine wastewater	3	− 6.9
	5	− 23.2
	7	− 20.4
	10	− 15.9

When the solution pH is equal to the catalyst’s pH_zpc_, the catalyst has a neutral superficial charge, while if the pH < pH_zpc_, there are positive charges in the catalyst’s surface, and for pH > pH_zpc_, the negative charges are predominant (Canle et al. 2017; Litter and Quici 2010). By analyzing the results of Tables 2 and 3, we can predict that adsorption will be favored when the effluent pH is 3 which will be confirmed in “Adsorption results for RM, RVR, and BVR”.

The materials were digested, and atomic absorption analysis was carried out to determine the amount of metals present in the volcanic rocks and in the red mud; results are presented in Table 4, with the aim of later being used as adsorbent or catalysts in the Fenton reaction.

**Table 4:** Catalyst’s characterization

Metal	RVR (%)	BVR^a^ (%)	RM (%)
Cu	0.003	0.010	0.002
Zn	0.004	0.005	0.001
Fe	4.266	5.576	5.38
Cr	0.139	0.002	0.055
Ni	0.091	n.a	0.041
Mn	0.051	0.022	0.005
Pb	0	n.a	0.004
Ca	0.435	0.199	2.538
Mg	0.986	2.004	0.086
Na	0.862	0.055	1.764
K	0.0460	0.057	0.272
Al	1.389	0.302	2.988

*n.a.* not analyzed

^a^Determined in Gomes et al. (2018)

Analyzing the results, it seems that the Al, Fe, and Mg are the main metals present in their constitution which may be promising for later tests in the Fenton.

### Adsorption results for RM, RVR, and BVR

**Fig. 1:**
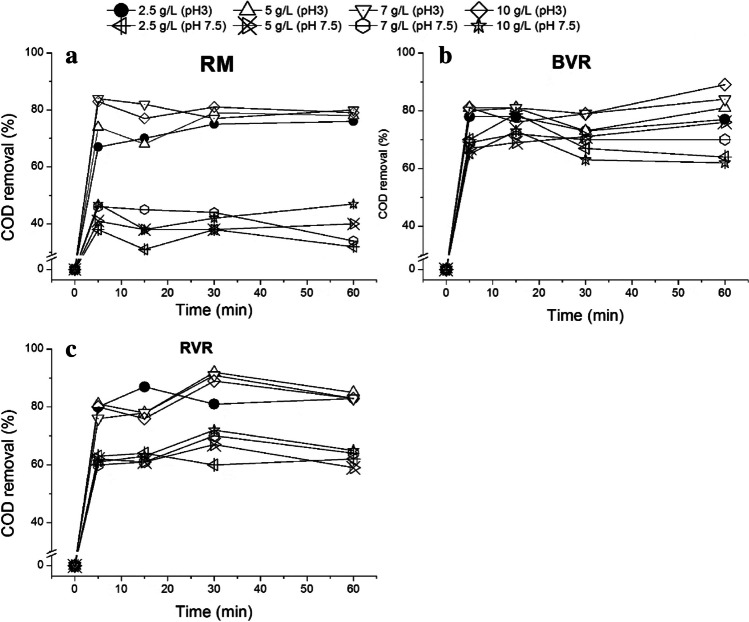
COD removal from diluted wastewater through adsorption with different materials (**a**) red mud (**b**) black volcanic rock, and (**c**) red volcanic rock as function of time

The pollutant adsorption was evaluated for the diluted wastewater, in which the results are presented in Fig. 1. A control test was carried out considering the effluent without any catalyst. Moreover, the pH effect (3 and 7.5 which is the natural pH of the effluent) and the adsorbent load (2.5–10 g/L) was evaluated. Besides, the iron fillings did not show any pollutant adsorption which is expected due to this material property (data not shown).

The catalyst’s adsorption capacity can be influenced by the catalyst’s pH_zpc_ and by the wastewater pollutant’s charge. Therefore, Table 5 evaluates the charges present in the catalyst and in the wastewater, which can be related to the adsorption results that were obtained.

**Table 5:** Charge’s evaluation in adsorption studies

Catalyst	Properties	pH = 3	Natural pH
RM	Catalyst charge	+	+
	Wastewater charge	−	−
	Interaction effect	Attraction	Attraction
RVR	Catalyst charge	+	0
	Wastewater charge	−	−
	Interaction effect	Attraction	No attraction
BVR	Catalyst charge	+	−
	Wastewater charge	−	−
	Interaction effect	Attraction	No attraction

+ positive charges, − negative charges, 0 neutral

The interaction between the catalyst surface and the wastewater charges can be the main responsible for the adsorption results. In fact, considering the RM results, at natural effluent pH, the adsorption capacity is higher which is expected since the diluted wastewater zeta potential is more negative under natural pH (*Ψ* =  − 19 mV at pH = 7) than under pH = 3 (Ψ =  − 10 mV). Moreover, considering the RVR experiments, the higher adsorption occurs for pH = 3, which may be related to the catalyst surface charge, since at pH = 3, the surface is positively charged, while at natural pH, the catalyst presents neutral charge. For the BVR, the adsorption behavior is interesting since at pH = 3, the rock presents higher adsorption values, but at neutral pH, COD reduction remains high. The adsorption is also related to the BET surface area (*S*_*BET*_).

Martins et al. (2013) estimated a BET surface area of 3.49 m^2^/g for the RVR, while Gomes et al. (2018) estimated a value of 28.3 m^2^/g for the BVR, and Domingues et al. (2019) calculated a surface area of 0.6 m^2^/g for the RM. Coupled to the charge’s interaction data, the catalyst’s *S*_*BET*_ can help to explain the reason why the best adsorption values were obtained for the BVR, followed by the RVR and the RM, respectively. The adsorbent load was studied in a range between 2.5 and 10 g/L; for further combination with the Fenton process, the adsorbent load was set at 2.5 g/L, minimizing the necessary load without compromising high COD removal. In fact, no important improvements in the adsorption efficiency are detected when comparing the performance of the materials at 2.5 g/L and 10 g/L load.

Another justification for the high performance of these materials in the removal of COD is that they are essentially composed of Al, Fe, and Mg, which are metals that are usually part of the coagulants or flocculants used in the effluent treatment. This kind of material promotes the precipitation of compounds in solution and the destabilization of colloidal suspensions of solid particles. Besides this, the volcanic rock materials present a high specific surface area.

### Fenton’s experiments

The Fenton’s process was carried to evaluate the capacity of the iron fillings in the swine wastewater treatment. Since this process is affected by several process parameters, the effect of the catalyst load and of the H_2_O_2_ dose was evaluated.

The Fenton process results from the reaction between a catalyst, usually iron, with an oxidant, hydrogen peroxide, producing hydroxyl radicals. When an excess of catalyst is present in the solution, it can scavenge the hydroxyl radicals (Babuponnusami and Muthukumar 2014).

The first approach to this study was based on the previous work of Domingues et al. (2021), in which the best conditions were [Fe^2+^] = 750 mg/L and [H_2_O_2_] = 750 mg/L for homogeneous Fenton process where iron sulfate (FeSO_4_) was the catalyst source. In this study, two approaches were made, one starting from the diluted effluent coagulated with polyDADMAC, such as in the work of Domingues et al. (2019), and the other starting from the real diluted effluent without any previous treatment step. Considering the above, the process optimization conditions were performed.

#### H_2_O_2_, catalyst load, and pH effect

**Fig. 2:**
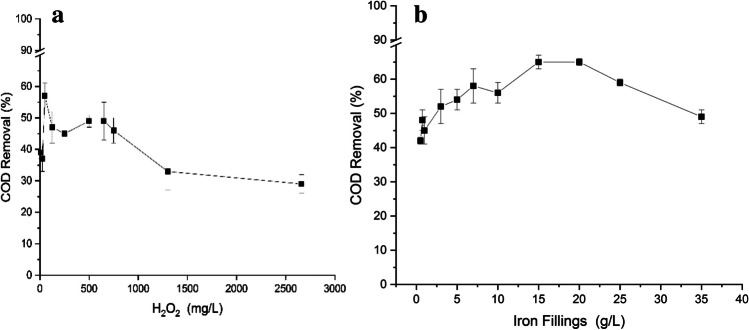
Evaluation of (**a**) H_2_O_2_ dosage effect in Fenton’s experiment with IF (coagulated effluent, [IF] = 5 g/L, pH = 3, *t* = 60 min) and (**b**)of IF load effect in Fenton’s experiment (coagulated effluent, [H_2_O_2_] = 50 mg/L, pH = 3, *t* = 60 min)

H_2_O_2_ and catalyst loads are two major factors determining process efficiency as well as operation costs. The optimization of the Fenton’s reagent is extremely important. The existence of H_2_O_2_ will help in the oxidation and in the consecutive reduction of iron. However, the H_2_O_2_ dosage must be investigated since this reagent can generate less reactive radicals (hydroxyl radical) or decompose into water when excessive quantities are used. Also, when it is present in lower doses, the performance of this technology decreases due to low reactive radical’s generation (Cheng et al. 2018). The efficiency of the Fenton reaction is impaired when the concentration of hydrogen peroxide is below or above its optimal value. Therefore, it is crucial to optimize the oxidant load. In this study, several doses of H_2_O_2_ were tested (10, 25, 50, 125, 250, 500, 650, 750, 1300 and 2660 mg/L), maintaining the IF concentration (5 g/L), pH = 3 during 60 min. The results are presented in Fig. 2a.

As already mentioned, the determination of the ideal catalyst load is also important because an excess load implies higher costs and can be adverse to the process, as it can promote the decomposition of H_2_O_2_ towards non-reactive oxygen species, and the scavenging of ·OH (Segura et al. 2013). For heterogeneous Fenton optimization, several iron filling loads (0.5, 0.75, 1, 3, 5, 7, 10, 15, 20, 25, and 35 g/L) were tested, during 60 min at pH = 3, considering the best H_2_O_2_ concentration of 50 mg/L. The results are presented in Fig. 2b.

Another relevant parameter in the Fenton’s process is pH; it is already commonly known and reported that the Fenton’s process present higher performance at acidic conditions (pH = 3). At neutral pH values, it is also possible to obtain degradation of organic compounds by heterogeneous Fenton, depending of the iron source applied, but the performance can be lower due to the presence of less reactive radicals (Rezaei and Vione 2018).

In terms of COD removal, Fig. 2a, the oxidant load that allows the highest removal of COD (about 60%) corresponds to 50 mg/L of H_2_O_2_. For higher values, there is a decrease in the COD removal efficiency, which means that an excess H_2_O_2_ load is harmful to the process.

In the previous studies, using the homogeneous Fenton, Domingues et al. (2021) analyzed the influence of H_2_O_2_ dosage, concluding that the COD abatement was comprised between 61 and 68% (best value for [H_2_O_2_] = 1000 mg/L). However, in this work, this profile is not verified. An increase in the hydrogen peroxide concentration showed a different behavior in the COD removal. The analysis to the degradation profile shows two maximum relative points (57% for 50 mg/L and 49% for 500 and 650 mg/L). In fact, these degradation profiles can be justified by the presence of the “scavenging effect.” This behavior can explain a decrease in the COD reduction when the hydrogen peroxide concentration is higher than 50 mg/L.

When the iron filling load was evaluated, the results showed a general increase in the COD removal until a concentration of 20 g/L; after this catalyst load, the degradation decreased. The increase in the COD abatement is expected, since at higher concentrations, there is more iron able to react and produce more quantity of reactive radicals. The decrease in the COD removal for higher concentrations of iron can be explained by the excess amount of this product; the excessive Fe^2+^ will react with the generated radicals and form Fe^3+^, consuming radicals that were necessary to oxidize the organic molecules. The results showed a higher removal of 66% for 15 and 20 g/L ([H_2_O_2_] = 50 mg/L).

In previous work, Domingues et al. (2021) showed that the increase of iron from 100 to 1000 mg/L led to an increase in the COD degradation (considering [H_2_O_2_] = 750 mg/L). For a [Fe^2+^] = 1000 mg/L, the authors obtained 72% of COD reduction. In fact, the homogeneous Fenton is, in general, considerably better than the heterogeneous Fenton. However, this work allowed to obtain a similar good performance (66% COD reduction in 60 min at pH = 3) involving heterogeneous Fenton, in terms of COD reduction, using a lower load of H_2_O_2_, which can considerably reduce the cost of the process.

Solid catalysts can have a lot of relevance in minimizing the great disadvantage of the Fenton process, which is the formation of iron sludge, presenting a low leaching of active cations. The IF presents the ability to promote a high H_2_O_2_ conversion with minimum decomposition, and they should be cheap, since it is a residue. It is considered that oxidation can occur either from iron ions released into solution, or through reactions between solutes and surface-species. As few iron ions are present in the aqueous phase, iron hydroxide/oxide formation can be avoided, and after catalyst separation, in some cases, the catalyst can be reused. The heterogeneous process has the disadvantage of being slower than the homogenous process because the target molecules must diffuse to the surface of the material to reach active sites before their degradation. On the other hand, solid catalysts have another great advantage, which is the possibility of its separation from the waste stream with a simple filtration. The use if iron wastes and a low amount of H_2_O_2_ (only 50 mg/L) is a positive point for the application of this process in the treatment of this wastewater.

Applying the Fenton’s process to the effluent at its natural pH (without lowering pH to pH = 3) caused a decrease in the COD removal from 66% (pH = 3) to 36% (natural effluent’s pH = 7.5). In the study of Domingues et al. (2021), when the pH was evaluated, the authors reached a maximum of 72% of COD removal considering [Fe^2+^] = 1 g/L and [H_2_O_2_] = 750 mg/L, in a 60-min reaction at pH = 3. When the pH was pH = 7, the authors obtained a COD abatement of 57%, for the same experimental conditions. It was proved that the grade of reaction between Fe^2+^ and H_2_O_2_ species is highly related to the solution pH. Under acid conditions, oxidation takes place by the reaction between H_2_O_2_ reacts with Fe^2+^ via the Fenton reaction to produce ·OH (Keenan and Sedlak 2008a, b).

At high pH, the precipitation of ferric hydroxide is favored and promotes the acceleration of H_2_O_2_ decomposition. Consequently, less ·OH are generated in the medium; thus, efficiency of Fenton reactions for the degradation of several compounds is reduced at very low and high pH values as well.

Comparing these studies allowed to show differences between the homogenous and heterogeneous Fenton, which are considerably higher for the natural wastewater pH (57% and 36% COD reduction for homogeneous and heterogeneous Fenton, respectively) than for pH = 3 (72% and 66% reduction for the same process order). Once again, these data also prove the good performance of Fenton (homogeneous and heterogeneous) under acidic pH (pH = 3).

## Integration of adsorption and Fenton

After understanding the adsorption capacity of the materials as well as the Fenton reaction capacity with IF, the next step was evaluating the combination of both systems to the swine wastewater treatment.

**Fig. 3:**
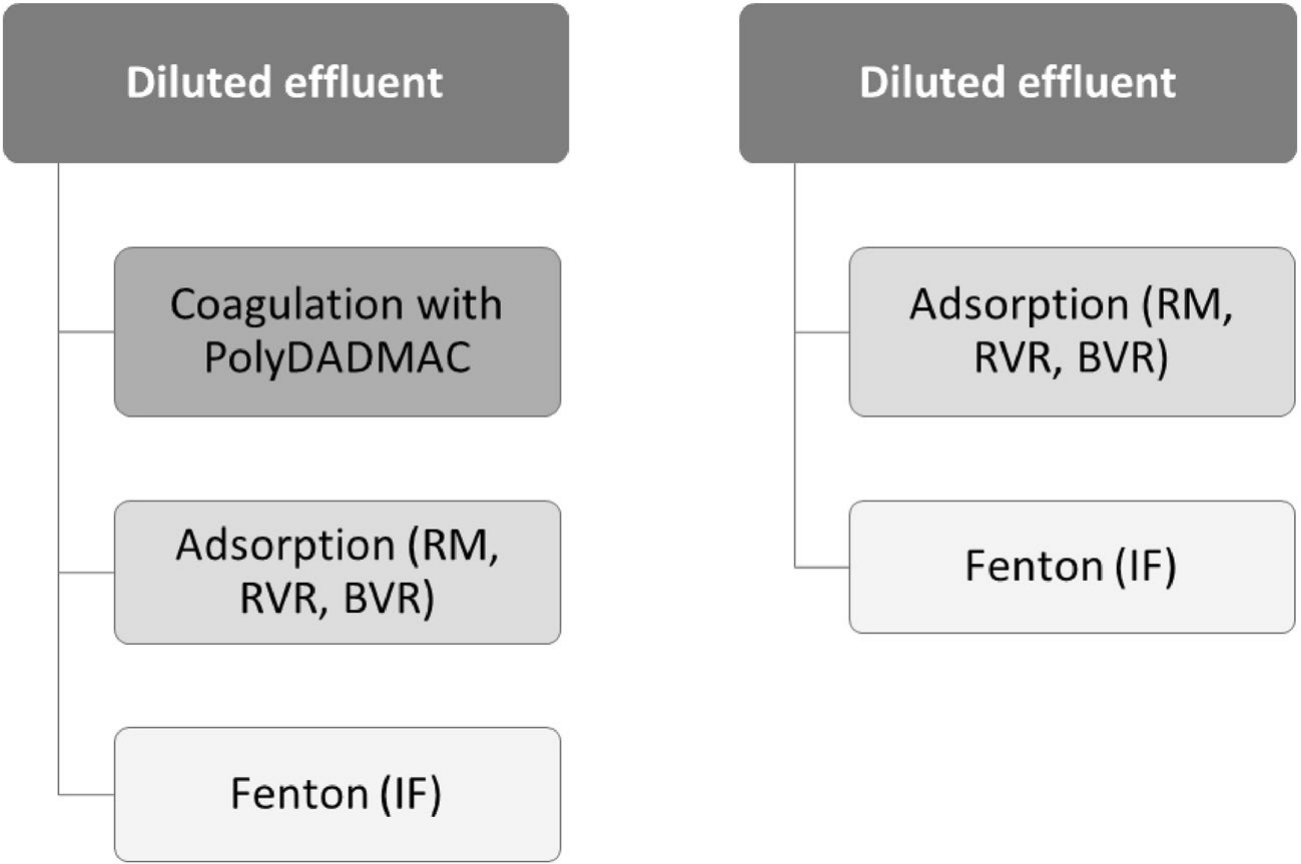
Representative scheme of the combined processes evaluated

These studies were carried for both coagulated and diluted wastewater. The adsorbents were used in the coagulated wastewater in the perspective of adsorbing contaminants that the coagulation process with polyDADMAC was not able to remove (Fig. 3).

When the diluted wastewater was considered, the perspective of these materials was to understand its efficiency in substituting the coagulation step with polyDADMAC. Table 6 summarizes the conditions applied in each experiment performed. As the minimum concentration studied shows good results in COD removal, it was fixed for all materials.

**Table 6:** Experimental conditions for adsorption and Fenton process

Adsorbent	Adsorption conditions	Fenton process conditions
RM/RVR/RBR	[ads] = 2.5 g/L	[IF] = 15 g/L
	*t* = 5 min	[H_2_O_2_] = 50 mg/L
	pH = 3	pH = 3
		*t* = 60 min

**Fig. 4:**
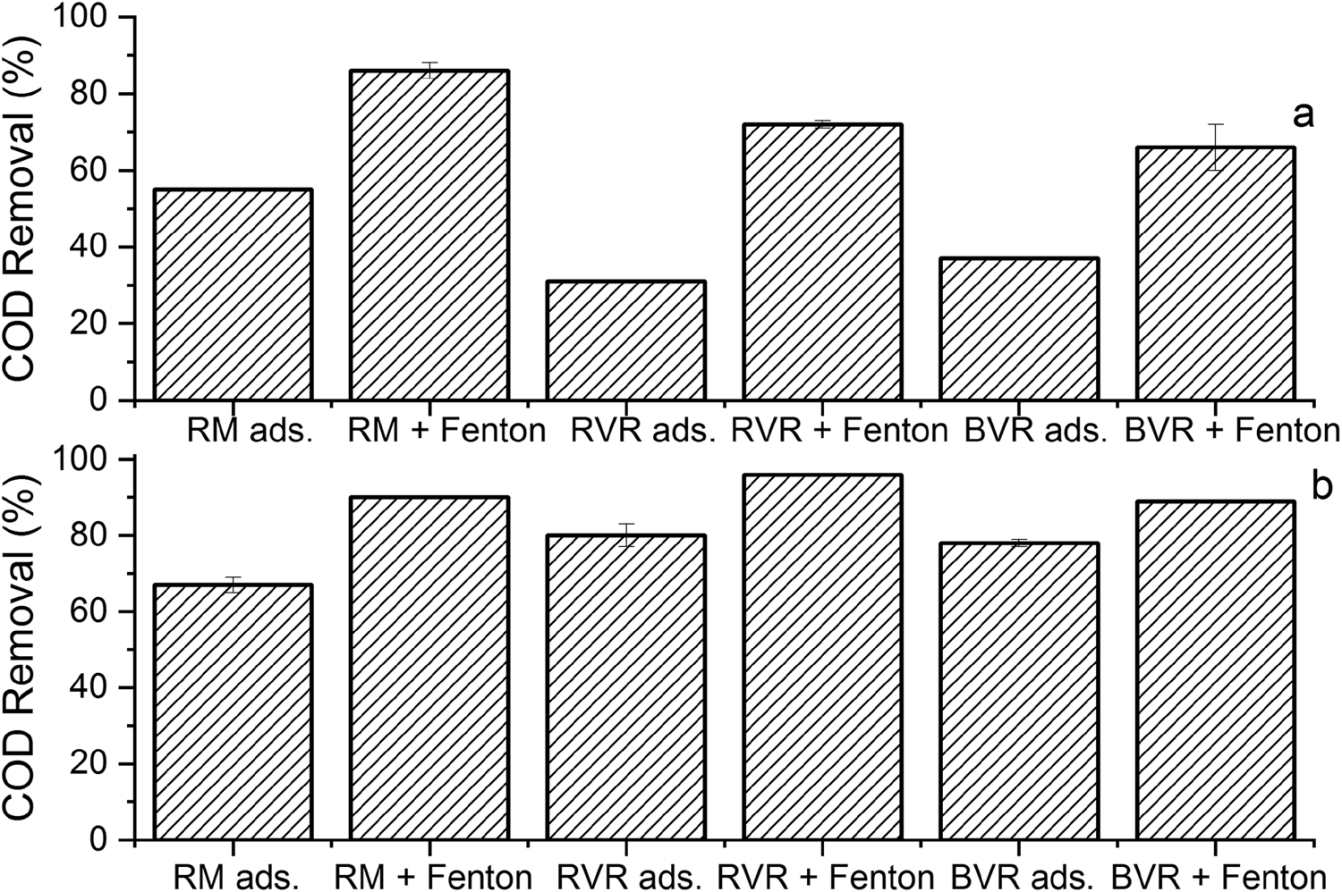
Adsorption and Fenton with IF for (**a**) the coagulated effluent (conditions mentioned in Table 6) and (**b**) the diluted effluent (operation conditions described in Table 6)

The results regarding COD removal are presented in Fig. 4a (for the coagulated wastewater) and b (for diluted wastewater), the adsorption performance of materials adsorbents alone (RM ads, RVR ads, and BVR ads) and the combination with Fenton process (RM + Fenton, RVR + Fenton, and BVR + Fenton).

In general, higher degradation results were obtained when the diluted effluent was considered and the studied materials (RM, RVR, and BVR) achieve greater COD reduction than using commercial coagulant polyDADMAC (~ 30% COD removal). This work allowed proving that the use of the studied adsorbents improves Fenton’s oxidation performance, and the coagulation step could be replaced. In terms of the COD discharge parameters (150 mgO_2_/L according to the Portuguese law DL 236/98), for the coagulated effluent, only the RM adsorption followed by IF Fenton allowed to obtain this value, while when the diluted wastewater is considered, more process combinations allowed to obtain this discharge value. A deep analysis of these parameters is carried in next section.

Regarding RVR and BVR adsorption efficiency, for the coagulated effluent, the COD removal was about 31–37%, while for the diluted effluent, this reduction increased until about 78–80%. In other words, the adsorption using the volcanic rocks allowed adsorbing a high amount of organic matter, which is quite impressive. Knowing that the coagulation process removes 30% of the organic matter, it is more favorable to use only the volcanic rocks than to use coagulation first with polyDADMAC followed by the rocks.

When the RM is used, the posterior application of Fenton allowed a good increase in the COD reduction for both wastewaters, but once again, higher performance is obtained when the diluted wastewater is treated by adsorption with RM followed by Fenton’s catalyzed by IF. For the coagulated wastewater, the RM adsorption led to 55% COD removal, and the posterior Fenton led to 86% COD removal (regarding to the wastewater initial COD value). For the diluted wastewater, these values were 67% and 90% for adsorption and adsorption followed by IF Fenton, respectively.

For the BVR, the use of coagulated wastewater allowed a decrease in COD of 37% and 66% for adsorption and adsorption followed by Fenton, while the diluted wastewater values were higher (78% and 89% for the same order).

The coagulants used frequently in the treatment of effluents are predominantly inorganic salts of iron and aluminum. When added to the effluent, iron or aluminum ions hydrolyze rapidly and, in an uncontrolled manner, form metal hydrolysis species. Materials based on either, aluminum or ferric ion, have been shown to perform better in some cases, in comparison with conventional coagulants such as aluminum sulfate (AS) or ferric sulfate (FS) (Jiang 2015). Since the materials studied have Al and Fe in their composition, this may be the explanation for the best performance in reducing COD when compared to polyDADMAC, which is a homopolymer of diallyldimethylammonium chloride.

## Analysis of treated wastewater

At the end of each process, the treated effluent was characterized in terms of COD, dissolved iron, TKN, and total phosphorous: Table 7.

**Table 7:** Treated swine wastewater characterization

Experiment	Type of wastewater	COD removal/[%]	Final COD value/[mg/L]	Iron dissolved in treated wastewater/[mg/L]	TKN in treated wastewater/[mg/L]	Total phosphorous in treated wastewater/[mg/L]
**Initial effluent**			**2980 -3150**	---	**245–273**	**72–80**
Fenton w/ IF	Coagulated effluent	66	352	0.98	43.59	16.7
RM adsorption		55	353	1.18	93.4	41.6
RM ads. + Fenton w/ IF		86	149	1.79	27.24	0
RVR adsorption		31	308	1.57	93.4	61.7
RVR ads. + Fenton w/ IF		72	297	6.50	105.08	18.8
BVR adsorption		37	480	2.54	101.18	53.1
BVR ads. + Fenton w/ IF		66	352	3.73	70.05	17.7
RM adsorption	Diluted effluent	67	293	7.15	140.1	79.9
RM ads. + Fenton w/ IF		90	131	1.81	---	3.2
RVR adsorption		80	345	3.56	70.05	71.9
RVR ads. + Fenton w/ IF		96	54	3.03	---	8.7
BVR adsorption		78	346	2.26	140.1	70.7
BVR ads. + Fenton w/ IF		89	148	2.76	---	10.2

--- not detected

The initial diluted effluent values are shown in bold, and the experimental conditions for adsorption and Fenton process are shown in Table 6

The Portuguese decree-law 236/98 establishes parameters of wastewater discharges. Among others, this DL established discharge values of COD = 150 mgO_2_/L, total Fe = 2 mgFe/L, total phosphorous = 0.5 to 10 mgP/L, and total nitrogen = 15 mgN/L. Other decree-law 119/2019 regarding the quality of water for irrigation establishes limits of total Fe = 5 mgFe/L, total phosphorous = 5 mgP/L, and total nitrogen = 15 mgN/L, also establishing other parameter limits.

According to the parameters analyzed in Table 7, we can verify that the process that presents the best framework according to the DL 236/98 and DL 119/2019 is the combination of sorption with RM followed by the Fenton process with IF. One should also bear in mind the low dissolved iron present in the solution after IF Fenton’s process (Table 7). This means that iron precipitation after Fenton’s process is not necessary to comply with legislation. Thus, IF Fenton’s process aids solving one of the major drawbacks of homogeneous Fenton’s process related to the iron sludge produced.

## Conclusions

Volcanic rocks and RM showed better performance as adsorbents allowing removals of around 80% in the case of rocks and 67% in the case of RM for the diluted effluent, while IF has a strong potential as a catalyst in Fenton reaction.

The combination of the adsorption process with RM followed by Fenton reaction, using IF as a catalyst, makes it possible to fulfill most of the requirements that establish the legal regime to produce water for reuse, obtained from the treatment of wastewater, as well as its use. The best results were obtained using adsorption with RM followed by Fenton with IF (131 mg/L of COD, 1.81 mg/L of iron and 3.2 mg/L of total phosphorous). Also, the effluent firstly coagulated with PDADMAC followed by adsorption with RM, and Fenton process with IF was in conditions for discharge or irrigation (149 mg/L of COD, 1.79 mg/L of iron). On the other hand, the application of these materials proves to be very interesting from a circular economy perspective, since these end-of-life materials can be useful in the wastewater treatment. Moreover, these processes have potential to be coupled with a biological treatment in a sequential approach. In the case of swine wastewater, adsorption and Fenton’s process can be applied after anaerobic and aerobic biological digestion to refine the treated water.

## Data Availability

The authors confirm that all data and materials are supported and comply with field standards.
